# Assessed and Emerging Biomarkers in Stroke and Training-Mediated Stroke Recovery: State of the Art

**DOI:** 10.1155/2017/1389475

**Published:** 2017-03-08

**Authors:** Marialuisa Gandolfi, Nicola Smania, Antonio Vella, Alessandro Picelli, Salvatore Chirumbolo

**Affiliations:** ^1^Department of Neurosciences, Biomedicine and Movement Sciences, University of Verona, Verona, Italy; ^2^UOC Neurorehabilitation, AOUI Verona, Verona, Italy; ^3^Immunology Unit, Azienda Ospedaliera Universitaria Integrata, Verona, Italy

## Abstract

Since the increasing update of the biomolecular scientific literature, biomarkers in stroke have reached an outstanding and remarkable revision in the very recent years. Besides the diagnostic and prognostic role of some inflammatory markers, many further molecules and biological factors have been added to the list, including tissue derived cytokines, growth factor-like molecules, hormones, and microRNAs. The literatures on brain derived growth factor and other neuroimmune mediators, bone-skeletal muscle biomarkers, cellular and immunity biomarkers, and the role of microRNAs in stroke recovery were reviewed. To date, biomarkers represent a possible challenge in the diagnostic and prognostic evaluation of stroke onset, pathogenesis, and recovery. Many molecules are still under investigation and may become promising and encouraging biomarkers. Experimental and clinical research should increase this list and promote new discoveries in this field, to improve stroke diagnosis and treatment.

## 1. Introduction

**Figure 1:**
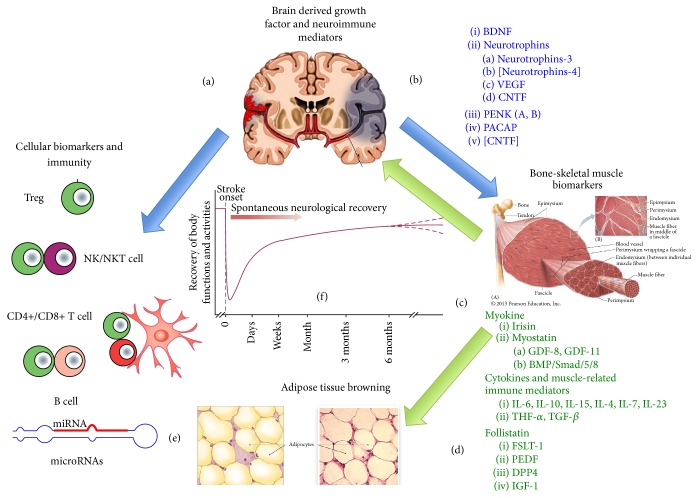
Cartoon showing the relationship between brain, muscle, and the immune system during stroke onset and stroke recovery, in order to highlight possible biomarkers of these events, as described in the text. Damaged brain, either following ischemic or hemorrhagic stroke, produces a panoply of different biomolecules, mainly BDNF or other neuromodulators, which link brain function with the immune system (a) and skeletal muscle (b). The first releases cytokines and cellular markers and the second one myokines, many of which interact with BDNF (c). Many of these myokines modulate the activity of other tissues such as vascular tissue (endothelia), bone, and adipose tissue. Irisin is a known “browning stimulator” (d). Downstream regulators of these mechanisms are represented by newly discovered miRNAs (e). The relationship between the activity of a defined biomarker (dose/time in plasma) and stroke recovery can be plotted in a time course curve as exemplified in (f).

Biomarkers in stroke have reached an outstanding and remarkable revision in the very recent years, since the increasing update of the biomolecular scientific literature in the field. Besides the diagnostic and prognostic role of some inflammatory markers, such as CRP, IL-6, TNF-*α*, or IL-1*β*, many further molecules and biological factors in the serum or plasma compartment have been added to the list, including tissue derived cytokines (myokines, adipokines), growth factor-like molecules, hormones, and microRNAs [1]. The latter ones have become important markers in many neurodegenerative and neuroimmune disorders, such as multiple sclerosis, Alzheimer disease, or Parkinson disease [2–5]. Neuroinflammation represents the main mechanism underlying the onset and development of stroke and the peripheral level of soluble immune factors and immune cells should give insights either on the onset and pathogenesis of stroke or on its recovery [6–8]. Poststroke rehabilitation, particularly following physical exercise and training, generates a crowded mass of mediators, more than 90, called myokines, which plays an emerging role in the biomarker field, which should update the role of plasma or circulating markers in stroke [9, 10] (see Figure 1).

Stroke risk and even poststroke recovery are strictly related to endothelial function. A correlation exists between arterial stiffness index and endothelia function in patients with acute ischemic stroke [11], while the association of stroke with hypertension should be better outlined. A recent paper reported that prestroke use of beta-blockers in hypertensive subjects did not affect neither stroke severity nor functional outcome [12]. The relationship between stroke and the cardiovascular system is particularly complex, as a huge panoply of different humoral and cellular participants make it highly complex to comprehend how stroke occurs and how to manage its recovery. Some recent papers revealed that the short-term management of hypertension in hypertensive patients has a positive effect on the long-term risk reduction of stroke [13]. Elevated arterial pressure remains a fundamental risk factor also for the pediatric population, in elderly subjects [14]. This would mean that a major concern for rehabilitation medicine and neurology is the search for the best circulating markers of this relationship. Many of these markers, actually, pertain to the ability of the immune system to counteract with the oxidative and mechanical stress associated with the cardiovascular function.

The most recent literature on the field stresses on the role of inflammatory molecules as biomarkers in stroke. Stroke, which can be simply considered as an injury occurring in brain when blood flow is cut off, may be of ischemic or hemorrhagic nature and each year about 800,000 people experience a new or recurrent stroke, being the fifth leading cause of death in the United States and about a third in Italy [15–18]. This circumstance suggests that searching for new emerging biomarkers for either stroke predictivity, diagnosis, or prognosis has come in the spotlight and is asking for new insights and data from experimental research [19, 20]. Emerging new biomarkers should come from the many novelties in the scientific field of stroke, which should help clinicians to improve addressing this pathology and its dramatic consequences also on the social life and habits. This review tries to give a state of the art of the topic.

## 2. Brain Derived Growth Factor (BDNF) and Other Neuroimmune Mediators in Stroke

The involvement in stroke of BDNF, a 13 kDa protein that belongs to the neurotrophin family, emerged some years ago. In particular low serum concentration of BDNF, particularly in the acute phase of ischemic stroke, is considered a factor of poor prognosis for the functional status of patients [57]. Physical exercise should ameliorate this circumstance, even by increasing the hippocampal level of BDNF in the early stages of a stroke (cerebral embolism) [58]. The enhancement of BDNF seems to be related to improvements in stroke recovery, even in animal models [59, 60], which show how social interactions are fundamental in the poststroke recovery [61]. BDNF increase in the hippocampus may improve also poststroke depression following estrogen-based therapy [37], a role that is also reached by physical exercise and muscular training [62]. Therefore, BDNF may be a good candidate to follow stroke development, even in chronic poststroke subjects, where circulating BDNF is very low with respect to controls [47, 48]. Besides BDNF further neurotrophins were recently involved in stroke and may fulfil the list of potential biomarkers.

Neurotrophin-3 has a major role in both traumatic and ischemic injury of the brain, such as in stroke. It is produced by neuroglia as an adaptation factor to hypoxic conditions and, together with proinflammatory cytokines such as IL-6 and TNF-*α*, it participates in the brain response to ischemic injury [49]. In mouse models, an upregulation of the neurotrophic receptor p75 (NTR) in striatal neurons during an ischemic damage was reported [40]. Neurotrophins should act with biochemical factors the ability of which is to regulated endothelial and vascular function. Actually, vascular endothelial growth factor (VEGF) has been recently associated with BDNF as a biomarker in stroke [63, 64], though with some criticism [39]. In animal models, the neurotrophin ciliary neurotrophic factor (CNTF), which is endogenously upregulated in a stroke onset, mediates the neurogenesis and an anti-inflammatory process [41]. The correct neuron-astrocyte interaction dampens CNTF release, which is upregulated by astrocytes therefore during a traumatic or ischemic damage to this integrin-mediated linkage [42]. To date there are no evidence reporting the role of plasmatic CNTF in stroke, though the circulating levels of this neurotrophin have gained much more importance in the study of patients with amyotrophic lateral sclerosis [43]. Still, in rat models, neurotrophin-4 increases its serum levels after a stroke event and exhibits the same properties of BDNF, as it is likewise a ligand of trkB [65]. Neuropeptide biology is moreover very rich of suggestions in order to retrieve emerging biomarkers for stroke. Yet, genetics should be involved in these issues, as genetic polymorphism highly influences the analytical performance of whatever would be introduced as a biomarker, particularly for neuropeptides. For example, neuropeptide Y may be a good prognostic biomarker in subjects with certain gene promoter polymorphisms [45]. Proenkephalin (PENK), or proenkephalin A, as proenkephalin B is known as prodynorphin, is a neuropeptide of recent introduction in the biomarker list. Its plasma level may be used as an indicator of prognostic outcome of stroke, as elevated concentrations in the bloodstream correlate with exacerbation of the cerebral injury [66, 67]. The neuropeptide pituitary adenylate-cyclase activation protein (PACAP) is involved, as many other factors here described, in the poststroke neurogenesis [68]. Also PACAP increase in plasma may be used as a prognostic marker of stroke, as an increase has been associated with severity of intracranial hemorrhage [69]. Neuromediators in stroke, detectable in the bloodstream and being considered as emerging biomarkers, should suggest that the ability to set a panoply of biomarkers for diagnosis and prognosis of stroke is a fundamental point at issue in the clinical research of this pathology.

## 3. Bone-Skeletal Muscle Biomarkers in Stroke

The involvement of the skeletal and muscular function, and its relationship, in stroke prevention and management, should be taken into account when addressing the issue of stroke biomarkers, as skeletal muscle undergoes deep changes in poststroke events [70]. Furthermore, myokines play a major role in the cross-talk bone-muscle [27, 71]. The role of skeletal muscle in stroke is of major interest. Markers of inflammation increase both systematically and locally in the skeletal muscle during stroke, while the anti-inflammatory feedback mechanism involves both myokines and the cholinergic anti-inflammatory pathway, which should be activated by physical exercise [9]. Myokines are muscle derived factors, with a role similar either to cytokines or growth factors, which modulate the complex relationship between skeletal muscle and other fundamental compartments, such as bone or adipose tissue, and whose upregulation is dependent on subject's physical exercise [21, 22, 27, 72]. Their activity in stroke should suggest a possible role as biomarkers of stroke pathogenesis and/or recovery [73].

### 3.1. Irisin

Irisin, otherwise known with its precursor name, that is, fibronectin type III domain-containing protein 5 (FNDC5), is a muscular trans-membrane protein, with a fibronectin type III-like ectodomain that can be cleaved giving the soluble molecule known as irisin [74]. This myokine fundamentally regulates the cross-talk skeletal muscle-adipose tissue [71]. Serum levels of irisin increase with training and physical exercise [75, 76]; therefore it may appear intriguing to ascertain if irisin may be involved as a possible and promising serum marker in poststroke training and stroke recovery or neuromuscular rehabilitation. To the best of our knowledge, there are no or very few reports showing or suggesting a relationship between irisin and stroke. During heart failure (HF), the expression FNDC5 was related to an improvement in the aerobic performance in HF patients, therefore suggesting that FNDC5 may work as a hormone counteracting stress coming from injury, tissue damage, hypoxia, and inflammation [77]. Its association with training is yet much more encouraging. Together with other myokines and neuromodulators, irisin should participate in the regulation of resistance training periodization, particularly in subjects with sedentary or rarely active life [78]. Furthermore, physical exercise induces the hippocampal expression of the brain derived neurotropic factor (BDNF), through a PGC-1*α*/FNDC5 pathway, that is, PGC-1*α* (which is a marker of mitochondrial function and biogenesis), during endurance exercise in mice, which elicits the expression of the neuronal gene for FNDC5, which in turn induces BDNF expression [79]. This relationship irisin precursor, namely, FNDC5, and BDNF may be of fundamental importance in the comprehension of the role of training in stroke [80], particularly because physical exercise induces BDNF but also synapsin I in the hippocampal trisynaptic circuit [81]. BDNF induces local synaptic plasticity [82] and more interestingly cyclin-dependent kinase 5 (Cdk5), a serine/threonine kinase involved in the rescue of synaptic plasticity [83], is involved in the BDNF-stimulated dendritic growth in hippocampus [84]. In poststroke patients, the role of Cdk5 has been associated with the long-term postischemic neurodegeneration and Cdk5 might be a pharmacological target; its inhibition or gene silencing increases the expression of BDNF in the hippocampal neurons [85]. The loss of this serine/threonine kinase in the nucleus accumbens reduces the ability to sustain a muscular physical exercise [85]. The relationship between the different degrees of the muscular activity (training, sustained and endurance physical exercise, moderate aerobic exercise, etc.) and the newly incoming biomarkers is still puzzling, particularly if related to poststroke rehabilitation. The role of irisin and its precursor FNDC5 in stroke recovery with training is quite far to be fully elucidated. As far as irisin is concerned, both muscle and brain express this hormone, which even participates in neurological and neuropsychiatric function such as regulation of behaviour and the mechanism of boosting reward-related learning and motivation [86] and is considered as one of the major linkers between muscular activity and brain [23]. Both irisin and BDNF are possible candidates as markers of sarcopenia [24], together with the transforming growth factor-*β* (TGF-*β*), follistatin, insulin-like growth factor-I (IGF-1), fibroblast growth factor-2 (FGF-2), osteoglycin, FAM5C, interleukin (IL-6), leukemia inhibitory factor (LIF), IL-7, IL-15, monocyte chemoattractant protein-1 (MCP-1), ciliary neurotrophic factor (CNTF), osteonectin, and matrix metalloproteinase 2 (MMP2), which affect also bone cells [27]. It is tempting to speculate that serum irisin would be an emerging biomarker in the relationship muscular activity/brain function and possibly an emerging biomarker also for stroke recovery.

### 3.2. Myostatin

This myokine, known also as growth differentiation factor 8 (GDF-8), is a member of the TGF-*β* protein family [25, 27] and has been recently related to the role and activity of GDF-11, with which it shares some similarities [25]. Myostatin is associated with muscle catabolism and actually antibodies against myostatin were considered to prevent sarcopenia, cancer cachexia, and muscle wasting disorders [26, 87]. People who survive stroke experience a disproportionate atrophy of their muscle mass or other detrimental tissue changes in the composition on the paretic side. Recent evidence supports the suggestion for a fundamental role of myostatin in these subjects, as an increase in myostatin mRNA was reported in the paretic thigh, while a reduction was observed following resistive training [88]. The serum level of myostatin, which is a negative regulator of muscle growth, has been associated with muscle function in a maintenance grip strength; that is, higher serum myostatin has been related to lower muscle function [89] and is a marker of muscle wasting [90]. Myostatin shares with irisin or FNDC5 a role in the browning phenomenon of the adipose tissue; therefore this myokine, as well as irisin, has a role in glucose and fat metabolism, besides muscle function [91]. This would suggest a possible relationship between stroke and nutrition in the myokine activity [92, 93]. Serum myostatin, as a possible biomarker in stroke-related disorders, has been reported for myocardial ischemia-related injury, as a cardiac myostatin upregulation immediately occurs after myocardial ischemia and participates in the ubiquitin-proteasome degradation of proteins, via the atrogin and MuRF1 involvement, in the skeletal muscle [94]. To the best of our knowledge, there are very few reports about the association of myostatin with poststroke neurorehabilitation, but evidence should suggest that this myokine may be upregulated following stroke and downregulated with muscular training. Animal models support this hypothesis. Muscle is involved in maintaining the bone mineral content and in electrical muscle stimulation following sciatic neurectomy in rats; muscle fibers downregulated myostatin gene expression, a model that should suggest the downregulation of this myokine in stroke-derived paretic limbs [95]. Cerebral ischemia causes also the activation of the bone morphogenetic protein (BMP)/Smad/5/8 signaling in muscle atrophy occurring following stroke. The ubiquitin-proteasome degradation of muscle proteins in paretic limbs following the severe sensorimotor deficits after cerebral ischemia involves a biomolecular mechanism in muscle fibers that inhibits the Akt/mTOR pathway and increases, besides myostatin, many actors of the ubiquitin-proteasome degradation such as muscle RING finger-1 or MuRF1, muscle atrophy F-box (MAFbx), and muscle ubiquitin ligase of SCF complex in atrophy-1 or Musa1 [96]. This evidence may suggest even a role of myostatin as a prognostic marker for stroke.

### 3.3. Cytokines and Muscle-Related Immune Mediators

Skeletal muscle is one of the major producers of interleukin-6 (IL-6), which contributes with other factors such as irisin to the fine regulation of bone metabolism and adipose tissue homeostasis after physical exercise [10, 97, 98]. The relationship between IL-6 and stroke is established principally by neuroinflammatory mechanisms in the CNS, where the expression of genes such as IL-6, besides myeloperoxidase (MPO), IL-1*β*, and TNF-*α*, is fundamental for stroke susceptibility [99] but also myocardial stroke generates a peripheral proinflammatory response in skeletal muscle [100]. In chronic heart failure training muscular exercise reduces muscle production of IL-6, TNF-*α*, IL-1*β*, and iNOS [101] although those markers involved in muscle atrophy, that is, atrogin and MuRF1, do not change their expression pattern in skeletal muscle [102], assessing that this model is not fully comparable to stroke-related muscle disorders. Following stroke huge panoply of proinflammatory cytokines that are released in the bloodstream and detectable in the serum, besides IL-6 and TNF-*α*, also IL-10, IL-4, IL-17, IL-23, and TGF-*β* increase [103]. Low frequency electrical stimulation together with acupuncture in denervation muscle induced atrophy in mice, reduced the expression of myostatin, and transiently increased the level of inflammation by enhancing the expression of IL-5, TNF-*α*, arginase-1 expressing macrophages (M1-type), and muscle specific microRNA, that is, miRNA-1 and miRNA-206, but also upregulated IGF-1 expression [104, 105]. This should suggest that inflammation in muscle is initially triggered to attenuate muscle degeneration and atrophy, by activating, for example, mitochondria-biogenesis markers, such as PGC-1*α* and autophagy [106–108]. Factors inhibiting autophagy in muscle fibers and the intracellular accretion of unfolded, damaged proteins may lead to apoptosis and muscle atrophy [109]. The intriguing relationship between muscle inflammation and PGC-1*α* is finely modulated. At least, as emerging from in vitro heart models, PGC-1*α* is upregulated following short-term exercise and interestingly an anti-inflammatory stimulus may reduce the activity of PGC-1*α* by attenuating its downstream effectors, such as NRF-1 and several respiratory genes, as most probably oxidative stress generated by either inflammation or muscular exercise is a main trigger of PGC-1*α* [110]. Mediators of this muscle response include several immune mediators besides IL-6. Interleukin 15 (IL-15) induces mitochondrial activity, via a PPAR-*δ* signaling during physical exercise [111]. Although there seems to be lack of evidence reporting a role of IL-15 in muscle atrophy following stroke, the most recent reports about this cytokine in this field suggest a possible involvement in this mechanism. At least, in diabetic rats, resistance training increasing both muscle and serum levels of IL-15 [112] and IL-15 is one of the main protective factors in sepsis-induced muscular wasting and proteolysis in mice [113]. In this sense, IL-15 should play a protective role against stroke or its dependent effects, as likely as further cytokines such as IL-19 [114] or IL-33 [115]. Despite its well-known anti-inflammatory role, IL-10 has been recently associated with worsening of muscle atrophy. Even a short bed rest in aged patients with leg lean mass or muscle wasting associated symptoms increases some proinflammatory cytokines and also IL-10 [116]. Furthermore, an excessive IL-10 response may even worsen stroke recovery, depending on genetics and sex [117, 118]. Actually, the inflammatory participation in stroke recovery should be profoundly revised. Interleukin 6 is still associated with worsening in muscle activity [119, 120], assessing the detrimental role of inflammatory cytokines in stroke-dependent muscle damage. But, as an example, anti-inflammatory drugs should act as a double-edged sword, both exacerbating brain injury and helping the pathway to poststroke recovery, suggesting the existence of more complex machinery in the neuromuscular rehabilitation [121]. For example, interleukin 17A (IL-17A), produced by *γδ*T, cells was initially thought to have detrimental action in the pathogenesis of acute ischemic stroke but a deeper focusing onto its activity showed that IL-17A participates in neuronal differentiation, synaptogenesis, and spontaneous recovery following ischemic stroke [122]. Both IL-17A and IL-23 failed in being associated with biomarkers in muscle damage, following physical exercise [123]. Fundamentally, muscle produces cytokines that are widely expressed in the innate and adaptive immune system, such as IL-6, IL-8, IL-10, IL-1ra, TNF-*α*, MCP-1, IL-1*β*, IL-12p35/p40, and IL-15 [124]. Particularly for IL-1 receptor antagonist (IL-1ra) it is well-known that this cytokine reverses immune suppression associated with stroke, generating concerns about the effect of immunosuppression during the acute phase of stroke [28]. Yet, there are controversial opinions about the therapeutic effect of this anti-inflammatory cytokine in stroke [29, 30]; probably one must distinguish the effect of systemic IL-1ra from local (e.g., skeletal muscle, myocardium). However, a lot of molecules with a growth factor-like activity have come in the spotlight as potential biomarkers in stroke.

### 3.4. Follistatin-Like 1, Insulin Growth Factors, and Other Myokines

Follistatin, known also as activin-binding protein (ABP), is considered as an antagonist of myostatin. Recent reports showed that the ratio myostatin to follistatin is a good marker of the denervated muscular atrophy and its recovery [125]. Circulating follistatin levels are correlated with muscular mass in healthy individuals [126]; therefore its presence in the peripheral blood should be interpreted as a positive prognostic marker of the recovery of muscle damage and atrophy following stroke, a hallmark of several myokines [73, 127, 128]. Follistatin-like 1 is a myokine which promotes revascularization and endothelial cell function following an ischemic injury [129]. Follistatin-like 1 (FSTL1) is a protein very similar to follistatin, which does not bind to activin A, but rather BMP4 and TGF-*β* [130, 131]. The neuroprotective role of FSTL1 has been reported in rats, where the glycoprotein is able to repair and improve neuron deficits inducing Akt phosphorylation and hence its receptor disco-interacting protein 2 homolog A (DIP2A) activation [132, 133]. FSTL1 is a marker of remodelling also in cardiac function, where in subjects with heart failure an increase in the serum FSTL1 was observed [31]. Actually, FSTL1 has been recently considered an independent circulating biomarker of inflammation and oxidative stress and likewise hsCRP, associated with markers predictive of stroke [32]. Despite its proinflammatory-like nature [130], FSTL1 is a cardioprotective molecule, which is upregulated following exercise training, particularly after myocardial infarction [134] and which modulates vascular remodelling [33].

Serum level of FSTL1 may give fundamental insights on the individual's response to ischemic stress. Pigment epithelium derived factor (PEDF) known as serpin F1, is a myokine with neurotropic activity, which has been recently associated, as a neuroprotective and antiangiogenic agent in animal models, with ischemic stroke [34, 135]. However, to the best of our knowledge, there are yet no data about the association between serum PEDF and stroke-related disorders, particularly for muscle. Rat models showed that PEDF induces the production of inflammatory chemokines such as MIP-2 and MIP-3*α* in microglia [136]. The myokine dipeptidyl-peptidase 4 (DPP4) has recently come in the spotlight because its inhibition, as well as the use of glucagon-like receptor 1 (GLP-1) agonists, leads to an antistroke effect [137, 138] and a cardioprotective role [139]. During physical exercise, DPP4 inhibitors improve mitochondrial biogenesis and muscle activity through the activation of GLP-1 signaling [140]. Yet, this myokine should act at a more systemic level, in the energetic balance of the organism, as their inhibitors are able to act in a similar way to sulfonylureas or pioglitazone for diabetes [141]. Insulin-like growth factors (IGFs), particularly with the involvement of CXCR4, are fundamental molecules in remodelling, even after stroke [50, 142, 143]. The serum level of IGF-1 in elderly men with muscle frailty has been considered as positive prognostic marker, also for bone mineral density [51]. IGF-I and IGF-II are important myokines recently related to stroke [52]. The relationship between IGF-1 and physical training is particularly intriguing, as serum total IGF-1 in response to a resistance exercise is highly variable and depends on the subject's body mass [144]. However, recent reports indicate that IGF-1 has a major role, together with BDNF, in neuroplasticity and in the recovery of the neuromuscular function following stroke by active muscle exercise [145–147], although an excess in IGF-1 production can induce neuroinflammation and exacerbation of stroke effects, as occurring following treatment with apolipoprotein A-1 mimetic peptide, which reduces white matter damage from stroke [148]. Therefore, besides the complexity of events related to IGF-1 activity, even when associated with BDNF, for many emerging myokines a possible role as biomarkers in stroke yet needs confirmation in clinical studies, despite the encouraging evidence coming from in vitro or animal studies. Usually, the serum level of IGF-1 in patients with stroke-derived intracerebral hemorrhage during admission (hospitalization) is lower than healthy controls, while VEGF and hepatocyte growth factor (HGF) are higher [149]. Further factors related to IGFs have been recently associated with stroke and may suggest emerging biomarkers in this pathology. A recent study showed that not only low levels of IGF-1 were associated with an unfavourable functional outcome of stroke but also the level of insulin-like growth factor binding protein-3 (IGFBP-3) [35]. More favourable outcomes should be yet associated with the evaluation of the decrease in the IGF-1 ratio and with IGFBP-3, rather than with serum levels of IGF-1 [150]. Actually, a more complex relationship between IGF-1, IGF-II, IGFBP-1, and IGFBP3 should give important insights on the risk of ischemic stroke [151, 152]. This complex pattern is a hallmark of many myokines involved in stroke. Myokines includes cytokines, hormone-like molecules, and growth factors. Fibroblast growth factor 21 (FGF21) is a myokine, which may be important in detecting subclinical atherosclerosis, which may be a pathogenetic cause of stroke [153]. FGF21 is related to metabolism, stress response, mitochondria function, and insulin resistance, as serum levels of FGF21 are elevated in certain types of mitochondria dysfunction, particularly in the muscle [154, 155]. Some reports have investigated also its relationship with bone physiology but the topic asks for further insights [156, 157]. It would be interesting to ascertain if serum level of FGF21 may be associated with positive outcome given by physical exercise. This has been reported for metabolic syndrome [158], yet not for poststroke training; then FGF21 may be suggested as an indirect biomarker.

Table 1 summarizes most of the assessed and emerging biomarkers in stroke onset and recovery.

## 4. Cellular Biomarkers and Immunity of Stroke

The role of the immune system in stroke and in its recovery-rehabilitation process, using physical training or others, includes both soluble factors (cytokines, chemokines, myokines, adipokines, and neuroimmunokines) and immune cells. Immune cells may be investigated mainly using flow cytometry and can give fundamental insights on the role and activity of innate immunity in the remodelling process following stroke [55, 56, 103, 159–161]. Some circulating cytokines and reactive molecules, such as interleukin 11 (IL-11), are considered important markers of ischemic stroke [162, 163], though some consideration should be taken into account about the role of the different subtypes of stroke [164]. Cellular signals, such as the immunoproteasome, are correlated with ischemic stroke with intracranial hemorrhage [165]. The phenotypic subsetting of the different lymphocyte populations would be an interesting biomarker of stroke and stroke recovery. For example, a peripheral persistence of CD4^+^CD28^−^ T cells (CD28* null* cells) has been reported in acute ischemic stroke [166], assessing previously reported evidence [167]. Fox P3^+^CD25^+^CD4^+^ regulatory T cells (Tregs) are presumably involved in stroke-related events [168]. In this regard, the program death 1 ligand (PF-L1) on Tregs should have a major role in neuroprotection against cerebral ischemia, as it mediated the suppressive action exerted by Tregs on metalloproteinase 9 (MMP9), which is released by inflamed neutrophils [169]. Innate immunity plays a fundamental role in stroke. It has been recently reported that nucleotide binding oligomerization domain- (NOD-) like receptors (NLRs), which are a class of cytoplasmic pattern-recognition receptors, are upregulated and highly expressed in a mouse model of ischemic stroke [170]. Recent pharmacological strategies have taken into consideration the innate immune hallmark of inflammation in stroke. This allowed researchers to realize that the anti-inflammatory mechanism induced by the damaged brain may be a good target for therapy. The cholinergic anti-inflammatory pathway, when modulated by *α*7-nicotinic acetylcholine receptor (*α*7-nAChR) ligands, may facilitate stroke recovery. By using a polychromatic flow cytometry approach, it is possible to investigate how circulating leukocytes change their surface phenotypes and subtypes and/or their amount in relation to stroke-associated-events. Invariant natural killer T cells (iNKT) infiltrate mouse ischemic hemisphere in animals undergoing an ischemic stroke [171, 172]. Alpha-galactosyl ceramide (*α*GC), which specifically activates iNKT, is requested to promote the protective role of iNKT in myocardial stroke [173], a circumstance that would be suggested also for brain stroke [172]. A high number of circulating NK cells within the first hours of an ischemic stroke, particularly if followed by a rapid falling down of other lymphocyte subsets, may indicate a possible risk of pejorative inflammatory disorders in stroke patients [174]. Infiltrations of NK cells in brain occur also in human during ischemic stroke, where cells are probably activated by IP-10 [175]. This evidence assesses the role of innate immune cells infiltration in the development of stroke-related damage. Stroke-induced lymphopenia is related to a reduction of circulating high mobility group protein B1 (HMGPB1) and by the activity of T cells [176]. CD4^+^ T cells, together with CD8^+^, *γδ*-T cells, and Tregs, change their peripheral pattern following stroke [177]. Very recently, Klehmet et al. reported that stroke induces defined alterations in the memory T cell compartment [178]. Gamma-delta T cells, which are with Th17 the main producers of IL-17A, increase dramatically during ischemic stroke [179]. Leukocyte subtypes that dynamically should change with stroke and change their surface markers are very different depending on the time of stroke onset and its subtype. Therefore, this evidence should render particularly complex any interpretation of the flow cytometry panel used to highlight the percentage and nature of the various lymphocyte subsets in the bloodstream. B cell compartment is also involved in stroke biology. Particularly, for pre-B cells, the released factor nicotinamide phosphoribosyltransferase (NAMPT), more simply known as pre-B-cell colony-enhancing factor (PBEF), plays a fundamental role in the mitochondrial survival and biogenesis after ischemic damage, protecting neurons from apoptosis [180]. B cells in stroke showed heterogeneity in their function and subtypes and participate in prestroke neuroprotective mechanisms [181]. Regulatory B cells contribute to limiting the inflammatory events occurring in CNS following stroke and IL-10 secreting B cells appear to have the major role in this mechanism [182]. Regulatory T cells have also a fundamental function in addressing stroke-related damage, particularly in poststroke recovery [183]. Actually, their role in this recovery process has suggested Tregs as a cellular therapy in stroke [170].

Much lesser importance has been given to circulating granulocytes in their possible relationship with stroke. Peripheral eosinophils have been associated with stroke, as the eosinophil count appears to have a fundamental impact on the outcome of stroke [184]. Blood neutrophil counts appeared to be associated with intracranial hemorrhage following stroke but this association was recently criticized [185, 186]. A role for basophils in stroke was reported several years ago but there is no further association to date, although mast cells are probably the early responders in the regulation, following ischemic stroke, of the blood-brain barrier [187, 188]. At least in mouse models, the CD36+ monocyte/macrophage system is involved in the poststroke recovery phase, leading to a correct phagocytosis [189]. In these models, monocyte-derived macrophages exhibit a repair function in the poststroke event [190]. Very recently, the role of monocytes in ischemic stroke has been thoroughly reviewed [191]. Interestingly, monocytes recruited to the ischemic site in mouse differentiate to an alternative activated macrophage (AAM) or M2-macrophage [56].

Particular interest has been recently devoted to brain dendritic cells in stroke events [192]. However, also antigen-presenting cells (APCs) in peripheral blood should give important insight on immune response to stroke and the mechanism of tolerance [193]. During cerebral focal ischemia a reduced peripheral costimulatory activity has been observed [194]. Stroke generates imbalance in the acquired immune response and a decrease in circulating dendritic cells [195].

## 5. MicroRNAs as Biomarkers in Stroke

MicroRNAs are the latest novelty in the emerging role of biomarkers in stroke [196]. These short modulatory RNA fragments play a fundamental role in the management of stroke, as much as that polymorphism in the microRNAs miRNA-130b, miRNA-200b, and miRNA-495 affects stroke susceptibility and the level of poststroke outcome [197]. MicroRNAs participate in the regulation of blood-brain barrier and in the function of microglia and astrocytes [198, 199]. Peripheral microRNAs are promising and emergent biomarkers for stroke [200]. Some miRNAs play prognostic or high diagnostic value to evaluate or predict stroke onset and development.

For example, low level of serum miRNA-320b is a high-risk factor for carotid atherosclerosis, a prodromic event possibly leading to cerebral ischemia and stroke [201], while miRNA-146a correlates with neuroprotection from cerebral ischemia [202]. The downregulation of miRNA-30a reduces ischemic injury via the enhancement of the beclin-1 mediated autophagy [203]. This neuroprotection role is exerted also by the downregulation of miRNA-181b, at least in the mouse model, via the involvement of the heat shock protein 45 and the ubiquitin carboxyl-terminal hydrolase isozyme L1, a role shared also by miRNA-30a [204, 205]. A neuroprotective role is exerted by miRNA-134 by targeting another heat shock protein, namely, HSPA12B [36]. Mesenchymal stem cells (MSC) may be primed by serum from stroke patients and this priming upregulates the expression of miRNA-20a, which in turn promotes MSC proliferation by regulating cell cycle and p21 CDKN1A [38]. This should suggest that miRNA-20a participates in the remodelling of damaged tissue after stroke. MicroRNAs as a biomarker for stroke may use either cerebrospinal fluid (CSF) or peripheral blood. After stroke some miRNAs, such as let-7c an miRNA-221-3p, are upregulated in CSF, while, in whole blood, where more than 250 different miRNAs were detected, miRNA-151a-3p and miRNA-140-5p were upregulated while miRA-18b-5p was downregulated [44, 46, 53, 54, 206–210].

## 6. Conclusions

Biomarkers in stroke represent a possible challenge in the diagnostic and prognostic evaluation of stroke onset and pathogenesis and in poststroke recovery. Many of the molecules described in the text are still under investigation and may become promising and encouraging biomarkers, either diagnostic or prognostic emerging biomarkers. In this perspective, research is actually asking for further insights, particularly about newly incoming myokines (for stroke recovery following muscular training) but also for those neuropeptidergic and neurotropic molecules, which should be better suited to fit as circulating biomarker in stroke rehabilitation due to nonmuscle exercise. Experimental and clinical research should increase this list and promote new discoveries in this field, in order to improve stroke diagnosis and treatment.

## Figures and Tables

**Table 1:** List of the main assessed and emerging circulating biomarkers in stroke.

Biomarker group	Molecule		Diagnostic or prognostic value^(1)^			References
Myokines	Irisin			**↑**	Good prognostic marker of stroke recovery with training	[21, 22]
	Myostatin (GDF-8)	**↑**	Muscular biomarker of stroke Muscle wasting			[23–26]
	Follistatin			**↑**	Good prognostic marker of stroke (muscular level)	[27–30]
	PEDF			**↑**	Good prognostic marker of stroke (angiogenic level)	[31, 32]
	DPP4			↓	Ameliorating stroke recovery	[33, 34]
	Osteonectin (SPARC)			**↑**	Neural repair following stroke	
	FGF-21	**↑**	Negatively associated with stroke			[35]

Neurotropic factors	Brain derived neurotropic factor (BDNF)	**↑**	Improvement in stroke recovery Biomarker of stroke onset	↓	Bad prognosis stroke recovery	[17, 19, 20, 36]
	Neurotrophin-3	**↑**	Biomarkers of stroke onset	**↑**	Stroke recovery	[37, 38]
	Neurotrophin-4	**↑**	Biomarkers of stroke onset			[39]
	CNTF	**↑**	Biomarkers of stroke onset			[40]

Neuropeptides	Neuropeptide Y			**↑**	Good prognostic biomarker in certain SNP patterns	[41]
	Proenkephalin A			**↑**	Bad prognosis in stroke progression	[42–44]
	PACAP			**↑**	Bad prognosis in hemorrhagic stroke progression	[45]
	Substance P			**↑**	Very bad prognosis in ischemic stroke progression	[46]

Growth factors and GF-like molecules	VEGF	**↑**	Biomarkers of stroke onset			[47–49]
	IGF-1, IGF-II			**↑**	Good prognosis in ischemic stroke progression (remodelling)	[50–52]

Cytokines	Interleukin 6 (IL-6)	**↑**	Stroke onset and progression	**↑**	Prognostic value to be reviewed	[1, 15, 16]
	Interleukin-33 (IL-33)	**↑**	Biomarkers of stroke onset	↓	Bad prognosis in ischemic stroke progression	[53]
	Interleukin 15 (IL-15)	**↑**	Biomarkers of stroke onset	**↑**	Brain injury	[54]
	Interleukin-11 (IL-11)	**↑**	Biomarkers of stroke onset			[55, 56]

^(1)^Arrows show the plasma and/or serum level or the level in peripheral blood.
